# Complete Genome Sequence of a *Brucella ceti* ST26 Strain Isolated from a Striped Dolphin (*Stenella coeruleoalba*) on the Coast of Italy

**DOI:** 10.1128/genomeA.00068-14

**Published:** 2014-03-06

**Authors:** Massimo Ancora, Maurilia Marcacci, Massimiliano Orsini, Katiuscia Zilli, Elisabetta Di Giannatale, Giuliano Garofolo, Cesare Cammà

**Affiliations:** a OIE Reference Laboratory for Brucellosis, Istituto Zooprofilattico Sperimentale dell’Abruzzo e Molise G. Caporale, Campo Boario, Teramo, Italy; b CRS4, Bioinformatics, POLARIS Scientific Park, Pula, Italy

## Abstract

*Brucella* spp. are important pathogens affecting a wide range of terrestrial and aquatic animals. We report the complete and annotated genome sequence of *Brucella ceti* ST26 strain TE10759-12, isolated from a striped dolphin (*Stenella coeruleoalba*) stranded along the Italian shoreline in March of 2012.

## GENOME ANNOUNCEMENT

*Brucella* spp. are facultative intracellular bacteria causing brucellosis, which is a worldwide-occurring zoonotic disease characterized by abortion in domestic animals and undulant fever, arthritis, endocarditis, and meningitis in humans. *Brucella* spp. comprise six classical (*B. abortus*, *B. melitensis*, *B. suis*, *B. ovis*, *B. canis*, and *B. neotomae*) and four novel (*B. ceti*, *B. pinnipedialis*, *B. inopinata*, and *B. microti*) species infecting terrestrial and aquatic animals (1). The genome of *Brucella* is composed of two circular chromosomes without any plasmids. An identity of >90% has been described among the genomes of the six classical species (1). Marine brucellosis has been recognized as a serious threat to seals, sea lions, walruses, dolphins, porpoises, and whales. Two *Brucella* species are recognized in marine mammals, including *B. ceti* from cetaceans and *B. pinnipedialis* from pinnipeds (2). However, cross-species infections may also occur, and zoonotic transmission cannot be ruled out (3). Since 1994, when *B. ceti* was first isolated from an aborted dolphin fetus, several cases have been reported worldwide (2). *B. ceti* strain TE10759-12 was isolated from a striped dolphin (*Stenella coeruleoalba*) stranded on the southern coastline of Apulia in 2012 (4). In recent years, with the implementation of sequencing strategies, more whole genome sequences of *Brucella* have become publicly available. However, except for scaffold sequences from three isolates (5), whole-genome sequences of *B. ceti* have not been available. Here, we announce the complete genome sequence of *B. ceti* ST26 strain TE10759-12, with the sequencing performed at the Istituto Zooprofilattico Sperimentale dell’Abruzzo e Molise G. Caporale in Teramo, Italy. This strain was previously characterized through multilocus sequence typing (MLST) analysis as belonging to the common sequence type 26 (ST26), while by multilocus variable-number tandem-repeat analysis (MLVA) of 16 loci, it was demonstrated to represent a genotype within cluster A (4). *B. ceti* ST26 is the most common *B. ceti* sequence type isolated from dolphins worldwide (2). Whole-genome sequencing of strain TE10759-12 was performed using an Ion Torrent PGM platform (~230-bp single-end library with ~20-fold coverage). The reads were *de novo* assembled using Velvet software version 1.1.0 (6). The genomes were finished by in-house-developed Python packages. Genome annotation was performed by Prokka, followed by manual inspection. The whole genome of strain TE10759-12 was demonstrated to have a G+C content of 57%, and it is composed of chromosomes 1 and 2, which are 2,117,718 and 1,160,316 bp, respectively. Furthermore, 9 complete rRNAs, 44 tRNAs operons, and 2,611 coding sequences (CDSs) were also described. In this report, the first complete genome sequence for *B. ceti* ST26 has been described, and it represents a crucial achievement for further studies on the evolution, epidemiology, and pathogenicity of this cluster of *Brucella*.

### Nucleotide sequence accession numbers.

The complete genome of *B. ceti* TE10759-12 has been deposited in GenBank with the accession no. CP006896 for chromosome 1 and CP006897 for chromosome 2.
